# Management Algorithm for Atypical Lipomatous Tumours: A Retrospective Case Series

**DOI:** 10.7759/cureus.77384

**Published:** 2025-01-13

**Authors:** Muhammad Umair, Maria Mahmood, Adeel Zafar, Amy Gillis, Paul Ridgway

**Affiliations:** 1 Department of Surgery, Tallaght University Hospital, Dublin, IRL

**Keywords:** algorithm, atypical lipomatous tumour, lipoma, well-differentiated liposarcomas, wide local excision (wle)

## Abstract

Introduction: Atypical lipomatous tumours (ALTs), or low-grade well-differentiated liposarcomas (WDLs), can be identified using radiological complex septations and histological atypia. In our view, this is a confusing name that underestimates the risk of local recurrence of such tumours. Defining a management algorithm for differentiating a lipoma from an ALT is important for considering the best management of these patients.

Aims: This study aims to evaluate the clinical, radiological, and histological features of ALT presentation and propose a management algorithm for these lesions.

Methods: A retrospective case series of a prospectively maintained database at Tallaght University Hospital, Dublin, Ireland was carried out on all patients with ALTs from 2013 to 2019. The group demographics, tumour characteristics, radiological features, treatment, and recurrence were described.

Results: From 2013 to 2019, 607 lipomatous tumours were resected; 40 lesions in 37 patients were classified as ALTs. The mean age of this subgroup of patients was 56.15 ± 13.64 years (range: 25-83 years). The most common location was the lower limb. All patients underwent clinical, radiological, and histological workups prior to surgery. Angio-embolisation prior to surgery was required in two (5%) patients; three (7.5%) patients developed local recurrence requiring a second surgical resection. Characteristics of ALTs and a management algorithm are proposed.

Conclusion: It is important for a practitioner to differentiate a suspected ALT from a lipoma. Increased intratumoural vascularity and septation in ALT are reflected in the MRI findings and may play a key role in the acquisition of a malignant phenotype in adipocytic tumours. The proposed management algorithm for these lesions aims to help stratify these subcutaneous lesions.

## Introduction

Lipomatous neoplasms are commonly encountered by the general surgeon as well as the surgical oncologist. Lipomas, the most common benign adipocytic tumour, are well-circumscribed, lobulated lesions comprised of adipose often separated from surrounding adipose tissue by a thin fibrous capsule that could occur on any part of the body [1]. If presenting intramuscularly, they can be poorly circumscribed and infiltrative, similar to well-differentiated liposarcomas (WDLs). Various other terms have been used historically in clinical practice and in previous literature to describe atypical lipomatous tumours (ALT), including pleomorphic liposarcoma, inflammatory liposarcoma, sclerosing liposarcoma, lymphocyte-rich liposarcoma, and spindle cell liposarcoma.

Differentiating large lipomas from ALT is challenging. While an experienced surgeon can frequently distinguish benign lipoma from aggressive liposarcoma, the nuances between large lipomas and similar atypical lipomatous tumours create a more challenging diagnostic dilemma. In the opinion of this research team, the term “atypical lipomatous tumour” is a confusing name that underestimates the risk of local recurrence of such tumours.

Fat cells (adipocytes) are the precursors for liposarcomas, most commonly found in the extremities and retroperitoneum [2]. A liposarcoma is one of the most common histologies of soft tissue sarcomas (STS), representing 50% of retroperitoneal and 25% of extremity STS [3]. Well-differentiated liposarcomas/ALTs are considered a low-grade malignancy that rarely metastasizes; they should be carefully followed as recurrence or dedifferentiation may occur [4]. Management with surgical excision and follow-up is still controversial because the recurrence rate is so variable, ranging from 0% to 69% [4-6]. This is further confounded by the fact that WDLs/ALTs frequently arise near major nerves or blood vessels [7]. Marginal resection seems to be the recommendation given that this may be adequate for these lesions to preserve critical structures [8].

A thorough literature review shows various studies proposing treatment strategies that would ensure the best possible prognosis and minimal risk of recurrence. It has been suggested in previous studies that sclerosing subtype and tumour rupture are unfavourable prognostic factors for local recurrence. Marginal resection is associated with a lower risk of tumour rupture than simple tumour resection, according to the Enneking classification. A preoperative core needle biopsy could be useful in identifying the sclerosing subtype.

Studies also suggest that extensive surgical resection of tumour tissue is a suitable treatment for all ALT/WDLs cases in order to avoid possible local recurrence. In addition, for ALT/WDLs tumours that are difficult to extensively excise, long-term clinical follow-ups are necessary due to the possibility of recurrence.

In the clinical setting, although differentiating a high-grade liposarcoma (usually solid, non-fatty density) from a benign lipoma is relatively easy, the distinction between deep-seated ALTs and lipomas is more subtle. The rate of growth and radiological and histological appearances of ALT and lipomas can overlap. The histopathological similarity between lipomas and ALTs has prompted the use of elaborate diagnostic techniques in routine practice for suspicious lipomas. In this study, we performed a retrospective review of patients diagnosed with ALT. The group demographics, tumour characteristics, radiological features, treatment, and recurrence were described. 

This article was previously presented as a meeting abstract at the 39^th^ Congress of the European Society of Surgical Oncology on October 9, 2019, with a change of authorship per the final contribution to the paper.

## Materials and methods

A retrospective cross-sectional analysis of a prospectively maintained database at a tertiary care hospital was carried out on all patients with ALTs from 2013 to 2019.

Study design

This study is a retrospective case-series analysis aimed at reviewing patients diagnosed with ALTs over a six-year period (2013-2019). The retrospective design allowed us to examine historical data to identify trends and outcomes, providing an in-depth analysis of clinical, radiological, and pathological features. A prospectively maintained database, the Hospital In-Patient Enquiry (HIPE) system, was used to identify patients with ALTs and their demographics. The pathology and radiology findings were analysed for further assessment and confirmation of the tumour pathology, size, and radiological findings. 

Study setting

The study sample was based at Tallaght University Hospital (TUH) in Dublin, Ireland. It is a 562-bed, 12-theatre hospital affiliated with Trinity College Dublin as an academic centre. Tallaght University Hospital is a specialist sarcoma management centre and the primary referral centre in Ireland. It, therefore, provides an ideal setting for this study, presenting an opportunistic sample. It allows access to a specialised and diverse patient cohort through an opportunistic sample.

Inclusion criteria

The study focused on patients diagnosed with ALTs who were managed at TUH between 2013 and 2019. Inclusion criteria required a confirmed diagnosis of ALT. The study excluded all other benign soft tissue lesions (e.g. lipomas) and malignant soft tissue sarcomas. These criteria ensured the study's scope remained specific to ALTs.

Data collection

Data collection was performed using the HIPE system, a prospectively maintained database that records clinical and administrative data on hospital admissions. This system provides comprehensive patient information, which enabled the study to access detailed records for all patients diagnosed with ALTs during the study period. The study employed non-probability consecutive sampling, meaning all eligible cases that met the inclusion criteria from 2013 to 2019 were included. This approach helped minimise selection bias and ensured that the study sample was representative of the ALTs managed at the hospital.

The information gathered during data collection included several key components. Demographic data such as age, gender, and any relevant baseline characteristics were recorded for each patient. Tumour characteristics, including tumour size, location, radiological appearance, and histological features, were noted to understand the clinical presentation of ALTs. Clinical and treatment data were also collected, focusing on the diagnostic imaging modalities used, the type of surgical interventions performed, and any adjuvant therapies administered to the patients. Lastly, outcomes data were captured, specifically addressing post-treatment local recurrence and other associated findings to assess the effectiveness of treatments and overall patient prognosis.

These data points were selected to provide a comprehensive view of ALT presentation, management, and outcomes. The study aimed to offer a thorough analysis of ALTs, enabling better understanding and potentially guiding future clinical decision-making.

Data analysis

The collected data were systematically entered into IBM SPSS Statistics software (IBM Corp., Armonk, NY, USA) for analysis. Quantitative variables, such as age and tumour size, were described using measures of central tendency (mean) and dispersion (standard deviation). Qualitative variables, such as gender, tumour site, and clinical features, were summarised using frequencies and percentages.

The dataset was stratified to examine correlations between clinical features, imaging characteristics, histological findings, and outcomes. Local recurrence rates were calculated to assess treatment effectiveness. Stratification also allowed subgroup analysis, such as evaluating outcomes by tumour location or recurrence in relation to treatment. The graphic representations were extrapolated using an online word cloud art creator, using WordArt.com (Saratoga, CA, United States) [9].

This study also aimed to identify patterns in ALT presentations and management that could inform future clinical guidelines. The study contributes valuable insights into a rare and challenging condition, offering an understanding of its management and outcomes within the Irish healthcare context.

## Results

From 2013 to 2019, 607 lipomatous tumours were resected. The mean age for these patients was 44.15 ± 8.55 years. A total of 483 (79.6%) were located in the torso (torso to extremity ratio was 4:1).

**Figure 1:**
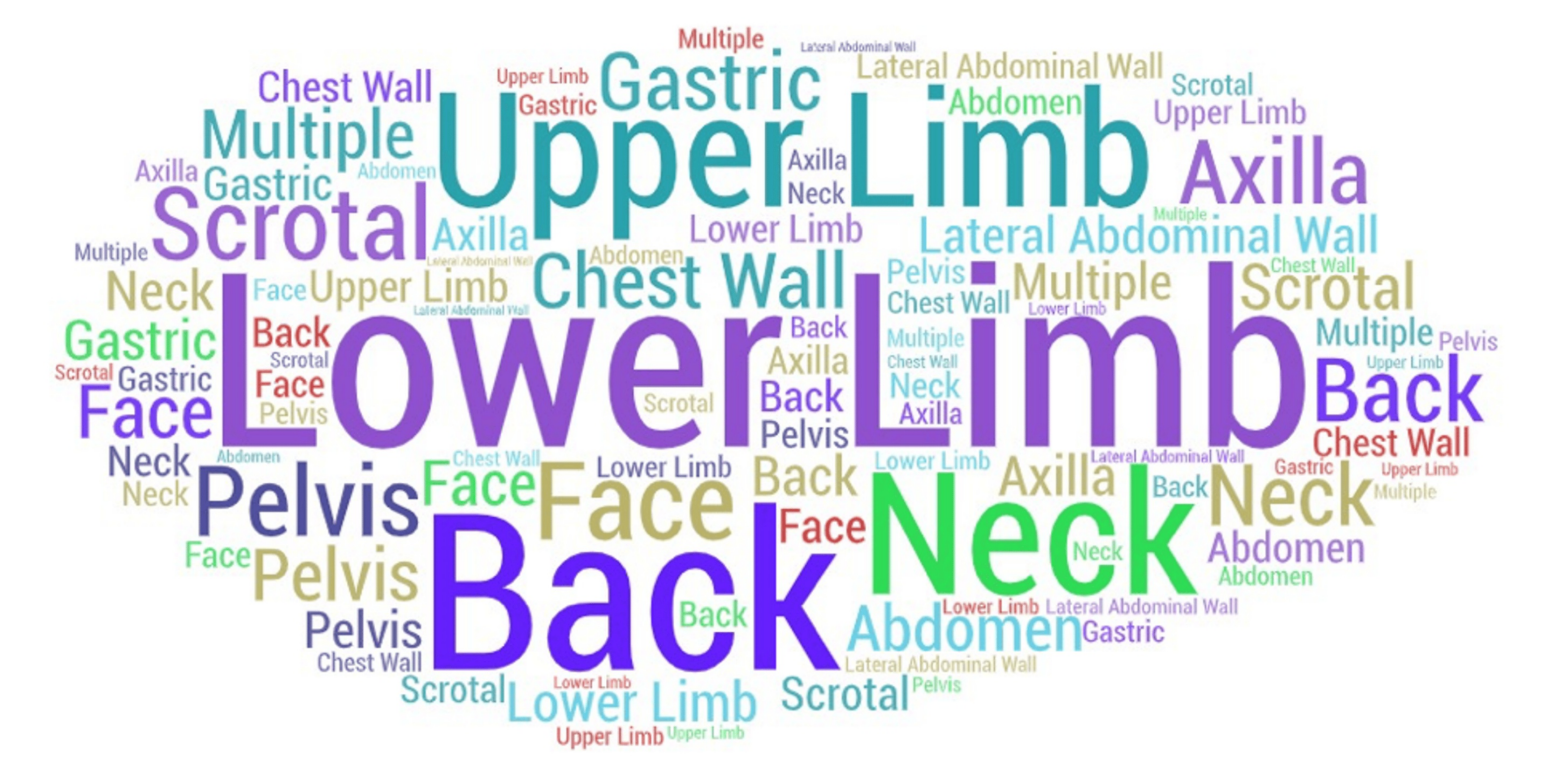
Site distribution of atypical lipomatous tumours in the study This graphic representation was extrapolated using an online word cloud art creator to represent the sample size of the visceral structures resected, respectively, according to their frequency. Created by the authors using WordArt.com [9].

Of these 607 tumours, 40 were classified as ALTs, identified in 37 patients. The mean age of this ALT subgroup of patients was 56.15 ± 13.64 years (range: 25 to 83 years). The mean size of the ALT subgroup specimens on histology was 9.05 ± 4.21 cm. Of these tumours in the ALT subgroup, 45% were found on extremities: lower limb (n=12) and upper limb (n=6). Other sites were the neck, pelvis, scrotal, axilla, chest wall, abdomen, back, face, lateral abdominal wall, and gastric (Figure 1).

In patients with certain characteristics and clinical findings, the lipomas were treated as suspected ALTs, and a complete clinical, radiological, and histological workup was performed prior to surgery.

In conclusion, individuals in the middle-aged adult group with larger-than-typical lipomatous and those who had recently noted changes in the size or consistency of the lump, or the lump was hard/firm in nature, if there was nodularity noticed in the lump, and/or there was a fixation to underlying structures, these patients should be further evaluated with additional diagnostic means. These sub-groups of patients should first undergo radiological imaging: ultrasound and/or MRI.

The imaging features of ALTs provide critical insights into their diagnosis and differentiation from benign lipomas and other soft tissue masses. A key characteristic is the significant fat content, which appears hyperintense on T1-weighted MRI and hypointense on fat-suppressed sequences. However, fat alone is not definitive. 

Atypical lipomatous tumours are distinguished by thick, irregular septations within the fatty matrix, contrasting with the thin, uniform septa typically seen in benign lipomas. These septations often enhance with contrast imaging, indicating increased vascularity and more complex tissue architecture, further highlighting their nature. 

**Figure 2:**
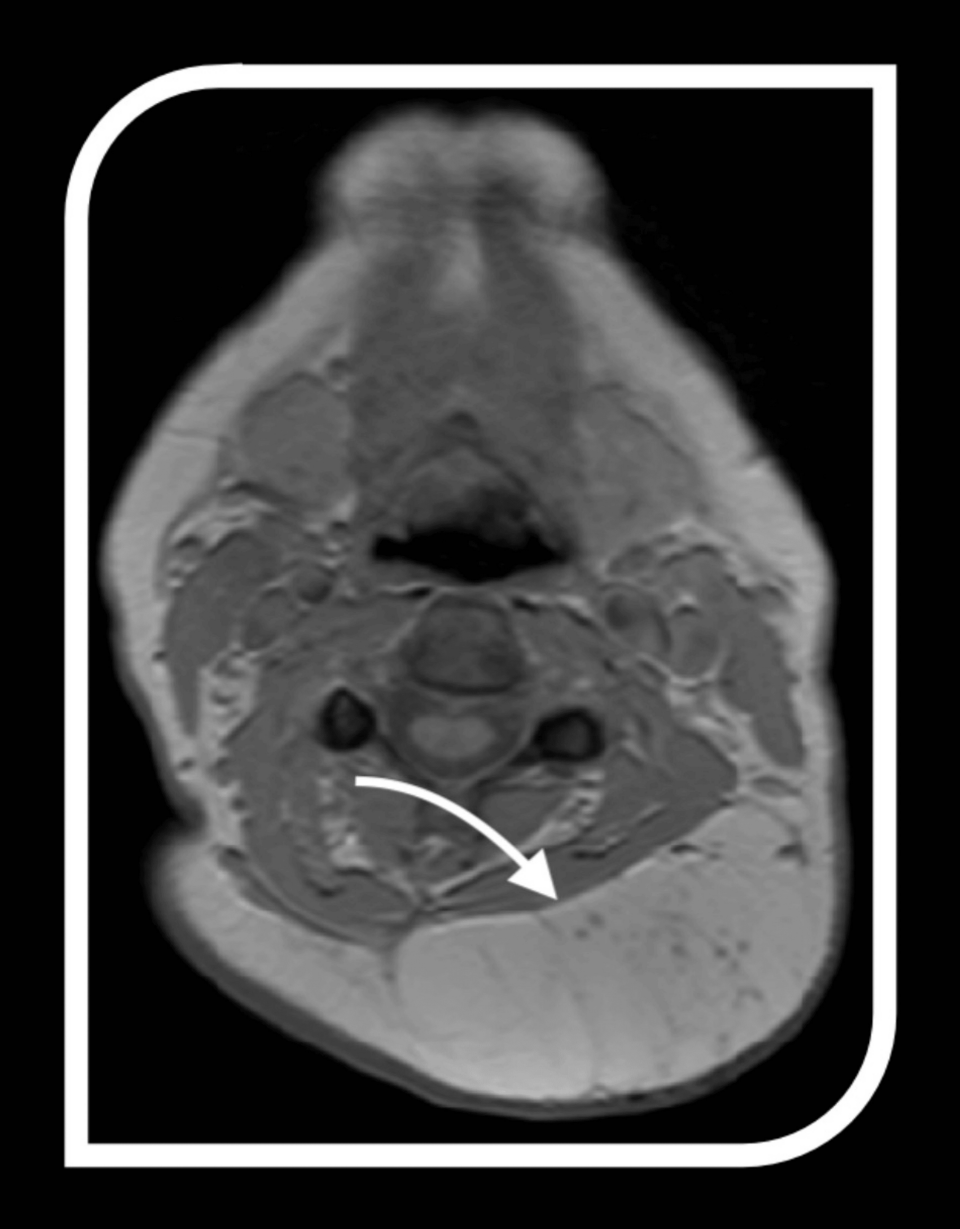
MRI imaging of atypical lipomatous tumours (ALTs) Representative features of ALTs on MRI employed in this study; the arrow indicates thick irregular septums and nodular contrast-enhanced areas.

**Figure 3:**
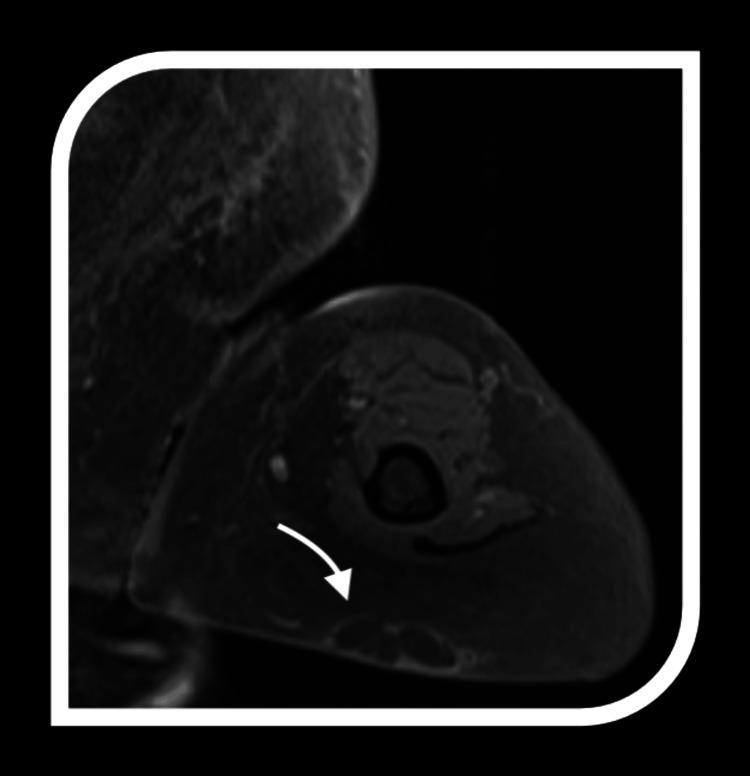
MRI imaging of atypical lipomatous tumours (ALTs) Representative features of ALTs on MRI employed in this study; the arrow indicates multiloculation, thick irregular septums, and nodular contrast-enhanced areas.

**Figure 4:**
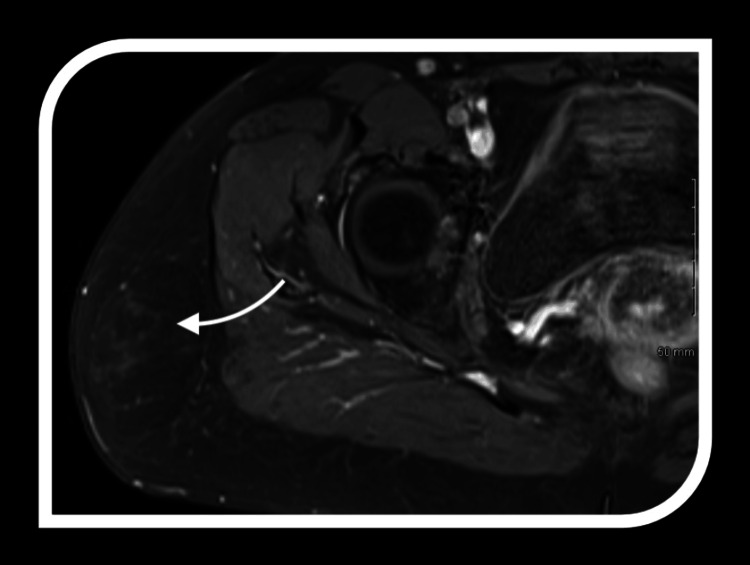
MRI imaging of atypical lipomatous tumours (ALTs) Representative features of ALTs on MRI employed in this study; the arrow indicates variable fat content, thick irregular septums, and nodular contrast-enhanced areas.

Another distinguishing feature is the presence of non-adipose components, such as fibrous tissue or soft tissue nodules, which appear as low signal intensity on T1-weighted imaging and intermediate to high signal intensity on T2-weighted sequences. These non-adipose areas may correspond to cellular atypia or dedifferentiation regions, adding to the tumour's complexity. Together, these imaging features provide a comprehensive framework for identifying ALTs and differentiating them from benign lipomatous tumours, aiding treatment planning (Figures 2-4).

**Figure 5:**
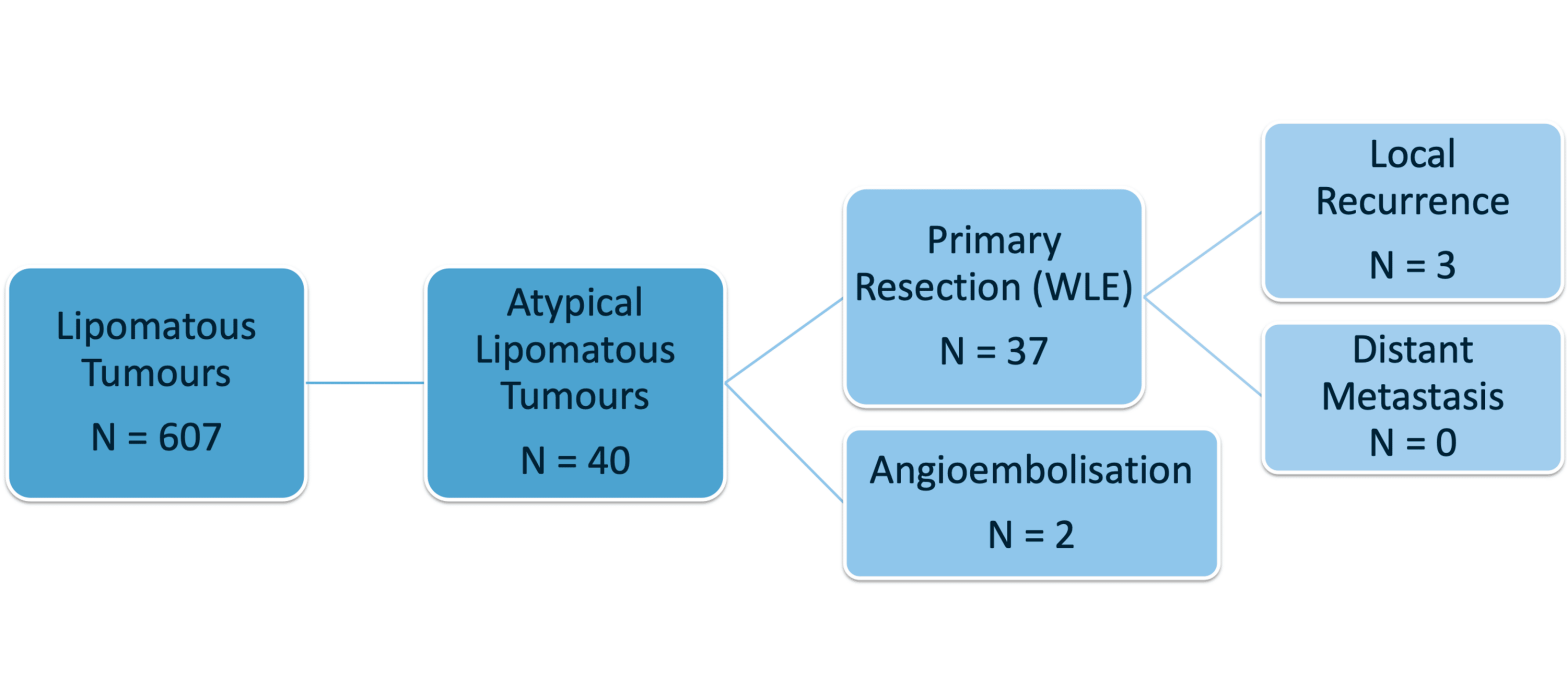
Case selection among lipomatous tumours in this study WLE: wide local excision

**Figure 6:**
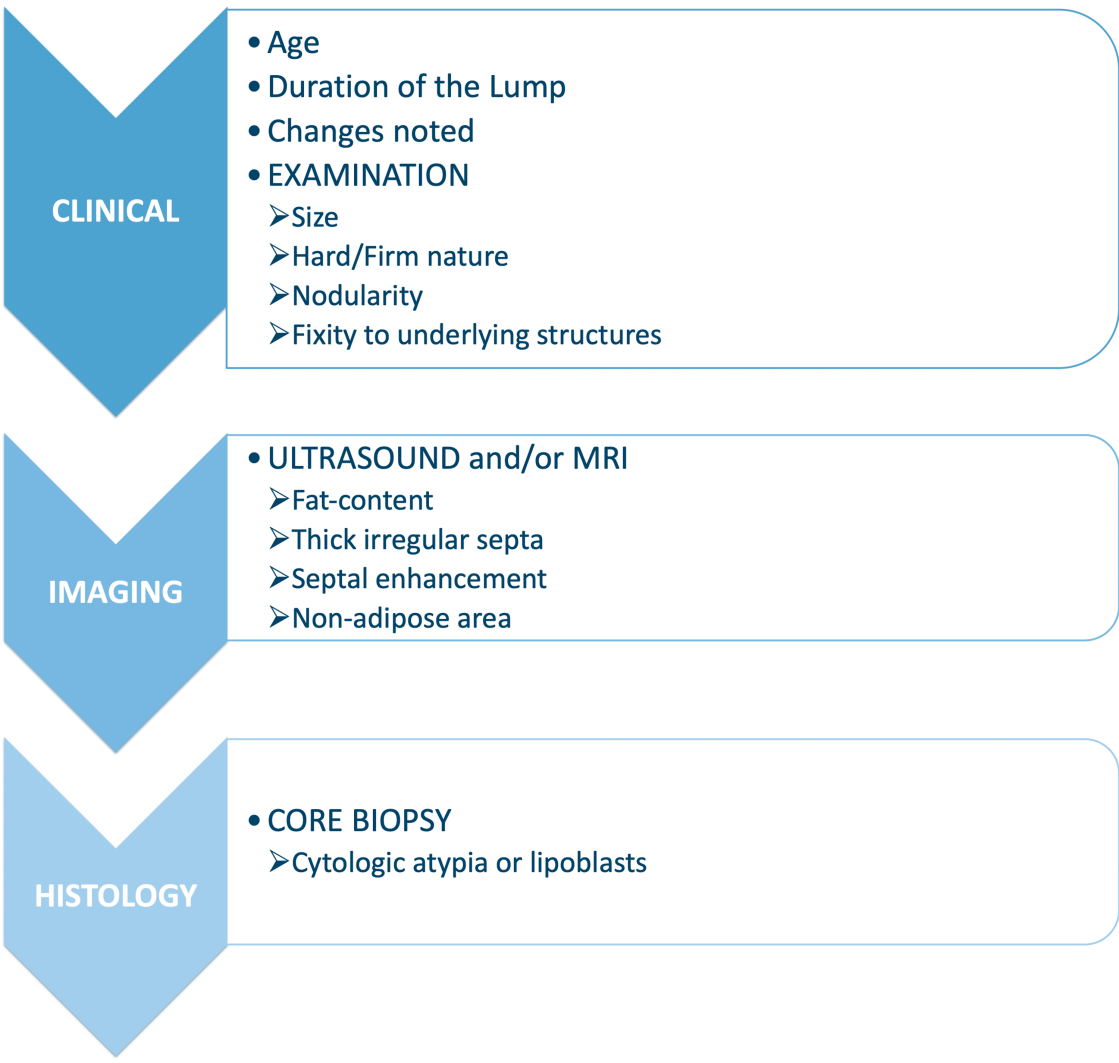
Diagnostic algorithm for atypical lipomatous tumours (ALTs) This diagnostic algorithm is based on the literature to differentiate ALTs from lipomas.

If certain suspicious features were found on imaging, then a core biopsy was performed. The core biopsies in these ALTs showed cytological atypia or lipoblasts. All 40 patients had primary resection with wide local excision; 5% (two patients) required angioembolization prior to surgery. Local recurrence occurred in three patients (7.5%), and they required a second resection (Figures 5-6).

## Discussion

**Figure 7:**
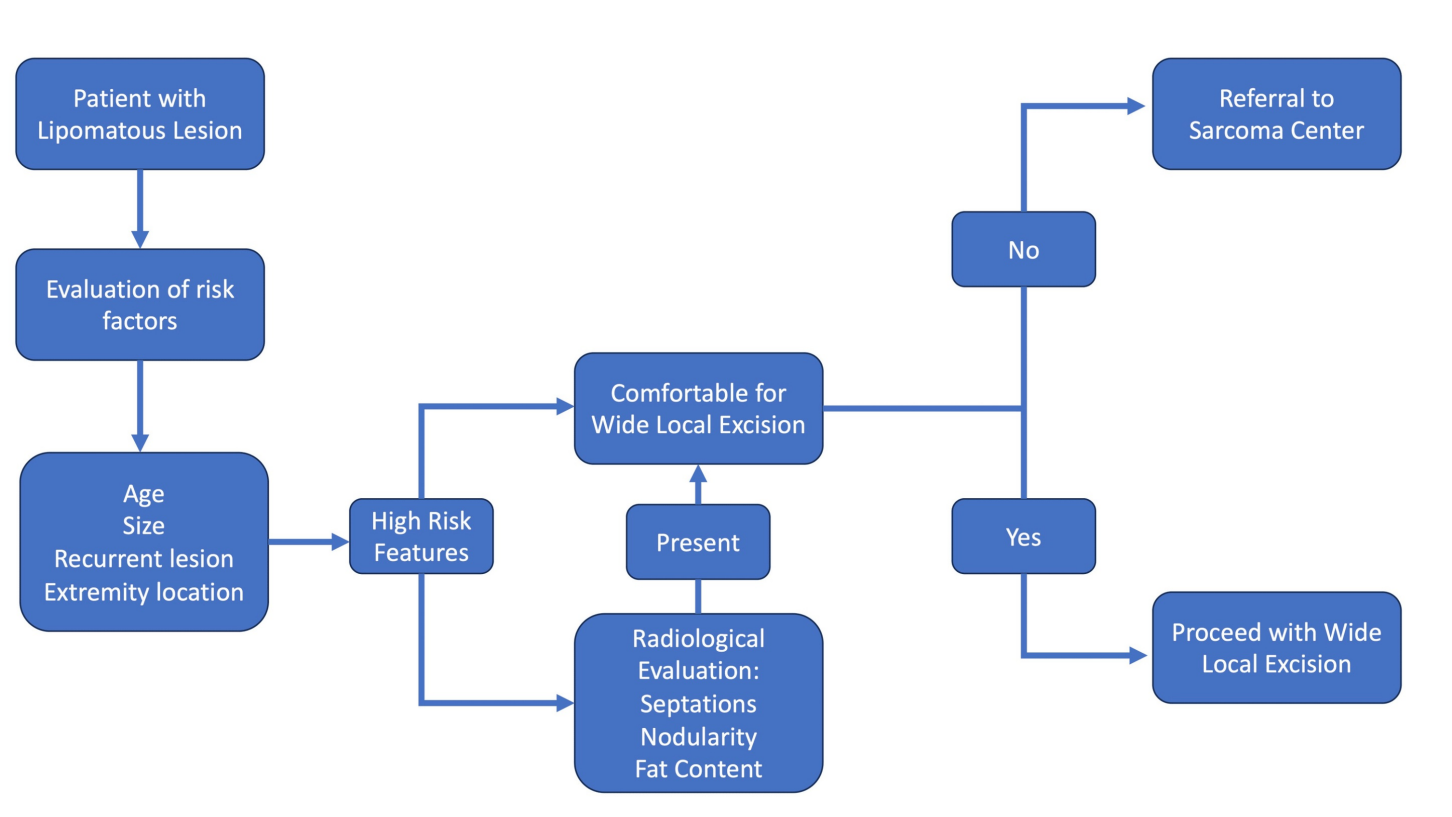
Therapeutic algorithm for atypical lipomatous tumours (ALTs) The developed therapeutic algorithm based on clinical parameters and MRI features to differentiate ALTs from lipomas.

The clinical similarity between a benign lipoma and an ALT presents a challenge for the surgeon to differentiate and stratify which patients might need a preoperative histological workup and imaging [10]. We propose an algorithm after observing the characteristics of ALTs in a tertiary care specialist centre, to make it easier for the clinician to identify patients that should go for pre-operative diagnostic workup (Figure 7). 

A preoperative biopsy confirming an ALT diagnosis would justify a slightly wider resection, rectifying the known potential for local recurrence after resection of ALT. Similarly, a biopsy confirming the diagnosis of a benign lipoma provides the advantage of avoiding an operation altogether for asymptomatic lesions, or marginal excision for the rest.

Marginal excision of ALTs outside the capsule of the tumour results in low local recurrence rates of approximately 10%, usually many years after primary surgery. The rate of dedifferentiation is very low and there are almost no reports of metastatic spread or disease-specific mortality. As a result, the surgery performed for both deep lipomas and ALT is often identical and only rarely involves radical resection. Previous studies also suggest both intramuscular lipomas and ALT showed a low recurrence rate when removed surgically from the extremities or trunk wall with intended marginal resection. It, therefore, seems safe to treat these tumours with marginal resection [11].

We found that most ALTs are on the upper/lower limbs, are in older patients, with nodularity and firm/hard consistency. Suspicious clinical findings should prompt preoperative radiological imaging and histological work-up, which may show one or all of the following features: thick irregular septa, septal enhancement, and non-adipose areas. Increased patient age and tumour size have been suggested by others as markers for ALT versus lipoma.

Characteristics of lipomatous masses that are associated with a diagnosis of ALT include patient age ≥55 years, tumour size ≥10 cm, previous resection, and extremity location (vs. torso). Similar findings were noted in our results. These easily identifiable traits may guide surgical management or referral to a specialist [10].

This study has certain limitations. Its retrospective design and reliance on the HIPE system may lead to potential biases. Being a single-centre study, the findings are not easy to generalise. The absence of a control group limits comparative analysis. Larger sample sizes, long-term follow-up data, and demographic stratification will be required to achieve external validity. However, the study can contextualise its findings and guide future research to address these gaps.

## Conclusions

Differentiating ALTs from benign lipomas is essential for practitioners due to their distinct biological behaviours, prognosis, and treatment pathways. Magnetic resonance imaging plays a pivotal role in this differentiation, with features such as increased intratumoural vascularity and prominent septation serving as key markers of ALT.

The proposed management algorithm provides a structured, patient-centred framework for stratifying subcutaneous lesions. Integrating clinical, radiological, and pathological findings, ensures that patients with ALT receive appropriate treatment. This approach not only optimizes patient outcomes but also improves resource utilization. Future research should focus on refining imaging criteria and exploring molecular mechanisms underlying ALT progression to further enhance individualized care.
